# Current status of day surgery nursing services and safety management in Hubei Province: a cross-sectional survey

**DOI:** 10.3389/fpubh.2026.1738645

**Published:** 2026-01-23

**Authors:** Zhihui Lu, Zijing Huang, Xiaobei Guo, Mengying Cui, Ying Wang

**Affiliations:** Department of Nursing, Tongii Hospital, Tongii Medical College, Huazhong University of Science and Technology, Wuhan, China

**Keywords:** day surgery, nursing management, nursing service, safety quality, standardized construction

## Abstract

**Objective:**

To investigate the current development status and nursing safety management of day surgery in medical institutions across Hubei Province, with the aim of providing a scientific basis for optimizing day surgery service processes and constructing a standardized nursing safety management system for day surgery.

**Methods:**

A convenience sampling strategy was employed to conduct a questionnaire survey among 205 medical institutions in Hubei Province from August to September 2024. The survey covered multiple dimensions, including basic information about medical institutions, the implementation status of day surgery services, the content of day surgery nursing services, and the index system for nursing safety management in day surgery.

**Results:**

Among the surveyed medical institutions, 66 (32.20%) offered day surgery services, with 72.73% being tertiary hospitals and 12.95% secondary hospitals. In terms of annual surgical volume, 87.88% of the institutions performed fewer than 3,000 day surgeries per year. Regarding nursing service content, the implementation rates of six key components did not reach 80%; among the 21 indicators in the nursing safety management index system, only seven had implementation rates exceeding 90%, eight ranged between 80 and 90%, and the remaining six were below 80%.

**Conclusion:**

Currently, the development of day surgery in medical institutions across Hubei Province is still in its nascent stages. There is a need to further increase the proportion of day surgeries among elective procedures, optimize the content of nursing services, and strengthen the standardized construction of nursing safety management systems. Simultaneously, the implementation of continuous nursing models should be promoted, and intelligent management tools should be utilized to comprehensively enhance the service quality and safety management levels of day surgery.

## 1 Introduction

Day surgery encompasses surgical procedures, including interventional treatments, conducted under anesthesia in designated day surgery units or operating rooms within inpatient departments. Patients are typically admitted and discharged within a 24-hour period in accordance with the treatment plan, excluding outpatient surgeries. In exceptional circumstances where extended hospitalization is medically necessary, the duration of the hospital stay should not exceed 48 h (1). Since its inception in European and American countries during the 1970s, day surgery has emerged as a widely adopted and mainstream surgical management model. This is attributed to its numerous advantages, such as enhancing the operational efficiency of healthcare facilities, reducing patient hospital stays, alleviating the financial burden of hospitalization expenses, conserving healthcare resources, and generating increased social benefits (2). In 2015, the establishment of the China Day Surgery Alliance marked a significant milestone, as the state subsequently introduced a series of policies related to medical insurance, infrastructure support, medical standards, and performance evaluation, thereby significantly advancing the development of day surgery services (3). In 2023, the National Health Commission introduced the “Action Plan for Comprehensively Improving Medical Quality (2023–2025),” which emphasizes the necessity of enhancing the organizational framework for quality management in day surgery to ensure patient safety. The adoption of day surgery represents an emerging trend in the advancement of public hospitals (4). Presently, day surgeries comprise over 60% of elective procedures in European and American nations, with countries such as the United Kingdom and the United States reporting proportions exceeding 85% (5). In contrast, day surgeries in China account for only 12.8% of elective procedures, suggesting that the practice remains in a nascent stage of exploration and development (6). This is compounded by challenges such as regional disparities in development and the absence of standardized information management and uniform service protocols (7, 8). Day surgery nursing service, as the core component of the overall day surgery service, is an integrated care process involving the participation of medical staff throughout preoperative evaluation, intraoperative cooperation, and postoperative follow-up. Existing national surveys mainly provide overview analyses at the macro-level. They lack targeting towards regional specific medical practices, and the research dimension of the core elements of day surgery nursing services is not refined enough. This makes it difficult to support the development of precise local-level quality control standards. In light of these factors, this research conducted a comprehensive survey to examine the processes, content, support systems, and quality management of day surgery nursing services in secondary and higher-level medical institutions across Hubei Province. The aim is to identify the weak links, thus aids in formulating regional standards for nursing services and safety management in day surgery settings within medical institutions, thereby promoting the enhancement of day surgery medical service quality and encouraging the efficient utilization of healthcare resources.

## 2 Subjects and methods

### 2.1 Subjects

Between August and September 2024, a convenience sampling method was utilized to administer a questionnaire survey across 205 medical institutions located in 13 prefecture-level cities/autonomous prefectures and three municipalities directly under provincial administration within Hubei Province. The questionnaire was distributed via online platforms, targeting day surgery administrators from the selected medical institutions as respondents. The inclusion criteria for the medical institutions were as follows: ① The institution must be classified as either a secondary or tertiary medical facility; ② The institution must have an operational surgical department with the necessary qualifications and routinely perform surgical procedures; ③ The institution must voluntarily agree to participate in the questionnaire survey and have obtained informed consent.

### 2.2 Methods

The questionnaire was meticulously designed by the Hubei Provincial Nursing Quality Control Center. It was developed based on an extensive literature review and in line with current clinical practices. It consists of three main sections: (1) basic information about medical institutions, (2) the content of nursing services for day surgeries, and (3) the safety management of day surgery nursing. The specific question design of the questionnaire is in line with its descriptive research objectives, focusing on collecting accurate and targeted factual information. It mainly includes three categories: binary questions (e.g., “Has the institution established a clinical nursing pathway for day surgery?” with options of “Yes/No”); multiple-choice questions (e.g., “Day surgery management mode” with options of “Centralized/Decentralized/Hybrid”); and fill-in-the-blank questions (e.g., “Nurse-patient ratio in the day surgery ward”). Based on this question type design, the questionnaire does not use traditional reliability and validity tests. Instead, it employs targeted quality control measures such as pilot surveys and data entry verification to ensure the scientific validity and reliability of the questionnaire.

The section on basic information encompassed various details, including the type and classification of the hospital, bed capacity, the management model for day surgeries, the number of operating rooms designated for day surgeries, the number of beds in day surgery wards, the nurse-to-patient ratio, the total number of day surgery cases, the top three surgical procedures conducted as day surgeries, and the rate of missed appointments.

The section concerning the content of day surgery nursing services encompassed various elements, including the establishment of a clinical nursing pathway for day surgery, pre-hospital management (comprising pre-hospital education and nursing assessment), in-hospital management (encompassing preoperative assessment and preparation, intraoperative nursing care, postoperative management and health education, and discharge nursing assessment and guidance), and post-hospital management (addressing the timing, frequency, content, and methods of follow-up care post-discharge), alongside the management systems and departmental configurations pertinent to day surgery.

The section on day surgery nursing safety management examined organizational management factors (such as system development, workflow, and risk prevention), environmental and facility considerations (including environmental layout and equipment), nursing staff factors (such as human resources and staff qualifications), and patient-related factors (including patient and family dynamics and their comprehension of health education content).

Research liaison officers were assigned based on the geographical location of the medical institutions. These officers were responsible for promptly resolving any ambiguities or questions that arose during the questionnaire completion process, verifying the content and logic of each item after submission, and keeping records for feedback. Regarding subsequent data quality control, two independent researchers conducted dual data entry, and any discrepancies were addressed to ensure the validity of the formal survey data.

Before starting the survey, the project team’s liaison officers contacted the nursing administrators at each hospital. After obtaining informed consent and before formally distributing the online questionnaire link, an online meeting was organized for the designated day-surgery administrators and research liaison officers of each institution. During the meeting, the research team communicated three core points: the survey’s fundamental objectives, detailed interpretations of the questionnaire content, and key filling precautions. This standardized guidance aimed to reduce response heterogeneity caused by inconsistent understanding of items across different institutions and improve data reliability.

Subsequently, the online link to the questionnaire was distributed to the nursing administrators of each medical institution, and the administrators in charge of day surgery operations were designated to complete the questionnaire for their respective institutions. To further ensure the quality of questionnaire responses, a pilot survey was conducted in advance among 30 day surgery administrators from 10 secondary and tertiary hospitals in Wuhan to test the clarity and operability of items. Based on the pilot feedback, ambiguous phrases were refined to avoid misinterpretation.

### 2.3 Data analysis

Data entry was independently conducted by two individuals utilizing Excel 2021, followed by a process of sorting and verification of the survey results. Any items or questionnaires exhibiting logical errors were excluded from the analysis. For partial missing data, only valid data were included in analyses, and missing rates were reported alongside results. Descriptive statistical analysis was executed using SPSS version 27.0. Normally distributed data are presented as mean ± standard deviation, while non-normally distributed data are expressed as medians. Categorical variables are summarized as frequencies and percentages.

## 3 Results

### 3.1 General characteristics of medical institutions

A total of 205 medical institutions across all 16 administrative divisions of Hubei Province were included. The sample comprised 36 Grade A Class III, 30 Grade B Class III, 87 Grade A Class II, and 52 Grade B Class II hospitals (In China’s hospital classification system, hospitals are first stratified by core function and service scope into classes (III, II, I, with III being the highest), and then graded within each class (A, B, C) based on comprehensive performance, with A representing the highest grade), with bed capacities categorized as ≤500 beds (*n* = 108), 501–1,000 beds (*n* = 69), 1,001–3,000 beds (*n* = 25), and 3,001–5,000 beds (*n* = 3).

### 3.2 Current status of day surgery practice

A total of 66 medical institutions offered day surgery services, representing 32.20% of all surveyed institutions. The provision of day surgery varied markedly by hospital tier, with 48 Class III hospitals (72.73% of all Class III hospitals) offering these services, compared to only 18 Class II hospitals (12.95% of all Class II hospitals). Regarding management models, 5 institutions (7.58%) utilized a centralized model, 50 (75.76%) a decentralized model, and 11 (16.67%) a hybrid model. Regarding annual procedure volume, data revealed that 58 institutions (87.88%) performed fewer than 3,000 day surgeries, 6 (9.09%) performed between 3,000 and 5,000, and 2 (3.03%) performed between 5,000 and 10,000. The most common surgical procedures performed in day surgery settings were circumcision (9 institutions, 13.64%), hysteroscopy and related procedures (9 institutions, 13.64%), cataract phacoemulsification with intraocular lens implantation (8 institutions, 12.12%), and ureteral stent removal (8 institutions, 12.12%). The median advance scheduling time for day surgeries was 24 h, and the median no-show rate was 2.50%. Collectively, these findings present a descriptive profile of day surgery availability, structural characteristics, and operational metrics, and do not identify determinants of its development.

### 3.3 Nursing staffing in day surgery

Staffing ratio data were available only from a subset of institutions that provided this specific operational information. The median nurse-to-patient ratio was 0.90 (IQR, 0.29–1.00), based on data from 17 medical institutions. Similarly, the median bed-to-nurse ratio was 1.00 (IQR, 0.40–2.50), as reported by 19 medical institutions. The proportional distribution of nursing staff was as follows: N1 level nurses: 0.25 ± 0.10, N2 level nurses: 0.39 ± 0.17, N3 level nurses: 0.29 ± 0.13, and N4 level nurses: 0.07 ± 0.08 (the N1–N4 classification is a tiered system used to categorize nursing personnel based on their clinical experience, specialized skills, and scope of practice, with N4 typically representing the highest level of competency). These values were derived from data provided by 11 medical institutions.

### 3.4 Nursing Service in Day Surgery

The nursing service for day surgery comprises three distinct phases: pre-admission, inpatient, and post-discharge management. The implementation rates for the following six service components were below 80%: pre-operative nursing assessment (family support assessment), post-operative health education (symptom self-assessment and sign monitoring), discharge nursing assessment (nausea and vomiting), discharge guidance (caregivers guidance), post-discharge management (emergency green channel protocols), and follow-up care (urination and defecation status, and psychological status). These gaps span the entire care continuum, from pre-operative preparation to long-term follow-up. Detailed data are provided in Table 1.

**Table 1:** Components of day surgery nursing services across 66 medical institutions in Hubei Province (*N* = 66).

Items	*n* (%)
Establish clinical nursing pathway for day surgery	
Yes	44 (66.67)
No	22 (33.33)
Pre-admission management	
Pre-admission education for day surgery	
Prehabilitation education	64 (96.97)
Relevant policy systems	61 (92.42)
Day surgery-related information	61 (92.42)
Perioperative medication guidance	61 (92.42)
Psychological nursing	61 (92.42)
Preoperative preparations for chronic and specialty conditions (e.g., eye drops, nasal drops, ear drops, antihypertensive medications)	59 (89.39)
Preoperative nursing intervention guidance for high-risk patients	55 (83.33)
Pre-admission nursing assessment for day surgery	
Health and physiological status (medical history, surgical history, medication history)	65 (98.48)
Review of diagnostic results (laboratory tests, imaging, electrocardiogram)	63 (95.45)
Mental and psychological assessment	55 (83.33)
Family support assessment	44 (66.67)
Inpatient management	
Preoperative assessment for day surgery	
Review of preoperative examination materials	65 (98.48)
Nursing assessments (e.g., fall risk, VTE risk, pain)	64 (96.97)
Identification of special circumstances arising during home waiting period	63 (95.45)
Preoperative preparations for day surgery	
Patient identification	66 (100.00)
Vital signs measurement	65 (98.48)
Implementation of physician orders	65 (98.48)
Special preparation (specialty related preparation)	58 (87.88)
Drug allergy testing	57 (86.36)
Intraoperative medication administration	57 (86.36)
Intraoperative nursing for day surgery	
Position management	66 (100.00)
Safety management	66 (100.00)
Vital signs and condition monitoring	65 (98.48)
Fluid management	62 (93.94)
Pain management	61 (92.42)
Relaxation techniques guidance	57 (86.36)
Postoperative management for day surgery	
Postoperative handover	64 (96.97)
Postoperative monitoring (vital signs, response to anesthesia, pain, catheters, wound)	64 (96.97)
Postoperative education	64 (96.97)
Risk reassessment	63 (95.45)
Adherence to Prescription Protocols and Delivery of Medication Education	63 (95.45)
Rehabilitation guidance	60 (90.91)
Postoperative health education for day surgery	
Dietary guidance	65 (98.48)
Wound care	61 (92.42)
Rehabilitation exercises	61 (92.42)
Medication management	60 (90.91)
Functional activity guidance	59 (89.39)
Follow-up planning and guidance	57 (86.36)
Safety recommendations	56 (84.85)
Positioning guidance	56 (84.85)
Symptom self-assessment and sign monitoring	49 (74.24)
Discharge nursing assessment for day surgery	
Wound and specialist symptom assessment	64 (96.97)
Capacity for action	62 (93.94)
Pain level	62 (93.94)
Self-care ability	58 (87.88)
Vital sign	58 (87.88)
Nausea and vomiting	51 (77.27)
Discharge guidance for day surgery	
Dietary guidance	65 (98.48)
Pain management	64 (96.97)
Wound care	63 (95.45)
Follow-up scheduling and procedures	63 (95.45)
Monitoring and management of abnormal conditions	62 (93.94)
Home medication guidance	62 (93.94)
Postoperative psychological support	57 (86.36)
Rehabilitation exercises	56 (84.85)
Caregiver guidance	51 (77.27)
Post-discharge management	
Post-discharge management for day surgery	
Specialized follow-up planning	60 (90.91)
The nurse guided the patient’s postoperative discomfort	57 (86.36)
Emergency access pathway (“green channel”)	51 (77.27)
Post-discharge follow-up for day surgery	
Wound assessment	63 (95.45)
Diet	60 (90.91)
Re-examination	60 (90.91)
Medication	59 (89.39)
Specialized related complications	59 (89.39)
Rehabilitation exercise	57 (86.36)
Urination and defecation status	48 (72.73)
Psychological status	48 (72.73)
Day surgery patients completed the first follow-up within 24 h of discharge	
Yes	52 (78.79)
No	14 (21.21)
Follow-up was conducted more than twice for patients discharged from day surgery, and the outcomes were duly recorded	
Yes	43 (65.15)
No	23 (34.85)
Follow-up methods for day surgery	
Telephone follow-up	65 (98.48)
Specialist follow-up	30 (45.45)
Internet + nursing services	10 (15.15)
Intelligent platform-based follow-up	9 (13.64)
Developing rehabilitation plans for day surgery patients	
Yes	43 (65.15)
No	23 (34.85)

### 3.5 Monitoring results of nursing safety management index system for day surgery

The day surgery nursing safety management index system comprised 21 indicators, which were assessed across 66 medical institutions, as illustrated in Table 2. While implementation rates exceeded 80% for most indicators, six priority indicators demonstrated rates below 80%. These deficient areas primarily concern structural and systemic foundations: the day surgery center access management system; standardized care pathways for specific diseases; the day surgery outpatient service center; the day ward and day surgery center are clearly demarcated, with safety signage that is highly visible and unambiguous; the electronic information system for day surgery; physiological indicators meeting standards for day surgery.

**Table 2:** Daily surgical nursing safety management index system monitoring of 66 medical institutions in Hubei Province (*N* = 66).

Items	*n* (%)
Presence of a day surgery nursing safety management organizational structure	
Yes	54 (81.82)
No	12 (18.18)
Presence of a day surgery center access management system	
Yes	52 (78.79)
No	14 (21.21)
Presence of day surgery nursing quality management standards	
Yes	55 (83.33)
No	11 (16.67)
Presence of a nursing assessment system	
Yes	56 (84.85)
No	10 (15.15)
Presence of a follow-up system	
Yes	61 (92.42)
No	5 (7.58)
Presence of standardized documentation and reporting norms for day surgery nursing	
Yes	54 (81.82)
No	12 (18.18)
Presence of defined job responsibilities and performance evaluation systems	
Yes	53 (80.30)
No	13 (19.70)
Presence of emergency protocols and drill systems	
Yes	62 (93.94)
No	4 (6.06)
Presence of a health education management system	
Yes	62 (93.94)
No	4 (6.06)
Presence of standardized day surgery processes for appointment, admission, examination, surgery, transport, handover, treatment, and discharge	
Yes	62 (93.94)
No	4 (6.06)
Presence of workflows for on-demand patient assessment, disease observation, and management of clinical changes	
Yes	62 (93.94)
No	4 (6.06)
Presence of a standardized care pathways for specific diseases	
Yes	32 (48.48)
No	34 (51.52)
Presence of terminal disinfection workflows for items used by patients with specific infections	
Yes	61 (92.42)
No	5 (7.58)
Nursing staff familiarity with day surgery risk points, hidden risks, and adverse event management protocols	
Yes	59 (89.39)
No	7 (10.61)
Presence of follow-up and referral management processes for day surgery patients	
Yes	53 (80.30)
No	13 (19.70)
Presence of an emergency green channel for day surgery patients	
Yes	58 (87.88)
No	8 (12.12)
Presence of a day surgery outpatient service center	
Yes	29 (43.94)
No	37 (56.06)
The day ward and day surgery center are clearly demarcated, with safety signage that is highly visible and unambiguous	
Yes	38 (57.58)
No	28 (42.42)
Real-time monitoring of operating room temperature, humidity, air quality, and surface environmental quality	
Yes	64 (96.97)
No	2 (3.03)
Presence of an electronic information system for day surgery	
Yes	32 (48.48)
No	34 (51.52)
Physiological indicators meeting standards for day surgery	
Yes	30 (45.45)
No	36 (54.55)

The most notable deficiencies cluster in several domains: (1) Integrated pathway management, including the lack of standardized care pathways and incomplete pre-operative/discharge assessments; (2) Infrastructure & system support, such as gaps in access management, dedicated outpatient centers, and electronic information systems; and (3) Post-discharge & safety continuity, encompassing follow-up care, emergency protocols, and clear safety signage.

## 4 Discussion

At present, the reach of day surgery remains limited, with only 32.20% of secondary-level and higher medical institutions providing day surgery services. In 87.88% of these hospitals, the annual day surgery volume is less than 3,000 cases. It is evident that there is considerable room for growth in terms of the number of day surgeries performed in medical institutions, and the proportion of day surgeries among elective surgeries should be increased. Concurrently, there remains a degree of polarization in the development of day surgery services. The adoption rate of day surgery in tertiary medical institutions is 72.73%, while that in secondary hospitals is only 12.95%. Within China, there is a pervasive trend of patients relying excessively on high-level hospitals, while neglecting to utilise primary health care services to the fullest extent (9). Promoting a hierarchical diagnosis and treatment system is proposed as a solution to the inadequacy of two-way referral mechanism (10). It is recommended to rationally allocate medical resources at all levels, improve the utilization rate of medical resources in secondary medical institutions, and address the structural imbalance in medical resource supply. In contrast to the centralized management model adopted by international medical institutions, the day surgery management model of medical institutions in Hubei Province is predominantly decentralized. Decentralized management is defined as the management of day surgery services handled by each respective department (11). Its management process aligns with that of elective surgery, and dedicated day surgery appointment and follow-up channels are established within each department. This model offers advantages in bed allocation and specialty-specific management but is not conducive to the standardized management of day surgery across the hospital (12). Building upon the decentralized management model that is predominantly adopted for day surgery in the current domestic context, we recommend its optimization within a homogenization framework. This can be achieved by implementing front-end management via a standardized day surgery procedure directory, enforcing clinical pathway management, simplifying medical record documentation, and establishing robust back-end quality control mechanisms (13).

The number and composition of nursing staff are critical to patient safety (14). This study analyzed the allocation of day surgery nursing staff in Hubei Province and identified two key findings. First, regarding the nurse-to-patient ratio, the median value was 0.90. While this figure aligns with common benchmark standards, it should be interpreted with caution as it is derived from a subset of institutional data. Second, in terms of competency composition, N2-level nurses constituted the highest proportion of day surgery nursing staff. Given that the N2 tier typically represents an intermediate level with relatively limited clinical experience compared to N3/N4 levels, this distribution may indicate a potential area for optimizing personnel structure within the current day surgery staffing model. In light of this structural consideration, we refer to the staffing framework proposed by Yu et al. (15), which recommends a competency-based configuration for specialized settings such as day surgery centers. Their model suggests an ideal composition of approximately 20% N4, 50% N3, and 30% N2 nurses, with no N1 nurses, aiming to enhance the capability of nursing teams in managing complex, high-turnover care without increasing overall staffing numbers. Adapting this structured, hierarchical approach to local contexts could serve as a strategic direction for improving the quality and efficiency of day surgery services.

Research shows that only 66.67% of healthcare facilities have developed clinical nursing pathways for day surgery. Among these, elements like pre-hospital family support assessment, guidance on relaxation techniques during surgery, postoperative psychological and rehabilitation guidance, self-assessment of symptoms and vital signs, and caregiver instruction are less frequently implemented. Studies indicate that patients undergoing day surgery have a low awareness of perioperative safety and generally experience high anxiety levels, which is associated with impaired postoperative recovery. The rate of postoperative complications has risen compared to traditional hospitalization (16). The brief duration of day surgery constrains the time available for effective communication between healthcare providers and patients, resulting in patients and their caregivers possessing inadequate knowledge regarding the disease, its symptoms, and perioperative precautions. This knowledge gap is closely linked to the aforementioned issues (17, 18). The “British Guidelines for Day Surgery” advocate for healthcare professionals to engage in direct communication with patients and caregivers, become acquainted with the surgical environment, and emphasize the assessment and enhancement of psychological and family caregiver capabilities (1). In response to the constraints imposed by limited time, Fang et al. (19) developed a comprehensive nursing checklist tailored for the entire day surgery process, concentrating on the three core aspects of “condition, needs, and outcomes.” By prioritizing patients’ psychological needs and experiences and implementing personalized nursing care, they successfully mitigated patient anxiety during the perioperative period of day surgery, reduced the severity of postoperative incision pain, decreased the incidence of postoperative complications, and enhanced patient satisfaction. Future research should focus on refining the critical components of the pre-hospital, intra-hospital, and post-hospital phases of day surgery. This entails addressing core needs such as patient psychology, family support, and perioperative information to enhance the quality of day surgery nursing and establish a comprehensive pathway for nursing services and health education. Additionally, explore accessible and scalable psychological and rehabilitation intervention models, leverage big data and artificial intelligence to construct personalized patient profiles integrating physiological and psychological characteristics, family caregiver capabilities, and social support levels. Based on these profiles, develop targeted interventions such as intelligent push of perioperative health education videos, AI-enabled postoperative symptom follow-up reminders, and online psychological counseling portals. This approach will realize precise delivery of nursing services, effectively make up for the insufficient communication time in day surgery, and further enhance the quality of personalized care.

Although the day surgery model presents benefits, such as enhancing the healthcare service framework, reducing hospital stays, and lowering hospitalization expenses, the markedly reduced in-hospital duration necessitates that patients undergo the majority of their recovery at home. This situation presents considerable challenges due to the limited professional knowledge and nursing skills of both patients and their caregivers (18, 20). Numerous studies have indicated that professional continuity of care services can deliver ongoing, tailored health education to patients recovering at home, thereby improving patient adherence and self-care capabilities, as well as encouraging the involvement of patients and their families in the care process (21, 22). The International Association for Ambulatory Surgery underscores the necessity for all healthcare institutions to ensure continuity of care for all day surgery patients (23). Our study indicates that 78.79% of medical institutions complete the initial follow-up within 24 h post-discharge for day surgery patients. However, only 65.15% of these institutions conduct more than two follow-ups and maintain comprehensive records for each discharged patient. Furthermore, 34.85% of medical institutions have yet to establish post-hospital rehabilitation plans for day surgery patients. These findings align with those reported by Fang and Yilin (24), suggesting that the continuity of care model for day surgery in Hubei Province is in its nascent stages and necessitates further development to adequately address patient needs. With the advancement of hierarchical medical systems, medical consortia, and related policies, coupled with the ongoing enhancement of community healthcare services, there is potential to develop an integrated hospital-community-family continuity of care model in the future. This model would involve the establishment of multidisciplinary teams, led by advanced practice nursing experts and guided by patient needs, to deliver personalized and comprehensive continuity of care services for day surgery patients. By utilizing a bidirectional referral system, community hospitals and medical consortium institutions can accept patients transferred from higher-level hospitals for preoperative preparation. Conversely, patients who require observation and symptomatic treatment without the need for specialized referral can be transferred to appropriate community health service centers or medical consortium alliance hospitals for ongoing observation and care. This approach not only ensures patient safety but also enhances the quality and efficiency of healthcare delivery. At present, follow-up methods for day surgery is still dominated by telephone follow-up and specialist follow-up. Considering the practical scenarios in medical institutions, a multi-modal follow-up strategy can be implemented. This strategy integrates intelligent platforms, telephone communications, official accounts, and secure social media channels, which adopt strict patient information protection measures for data security. It can establish seamless online consultation channels, ensuring the accessibility of health information while protecting patient privacy. Thereby ensuring the accessibility of health information. Through remote interaction methods such as virtual rehabilitation technology and wearable devices, continuous monitoring, dynamic follow-up, and precise guidance can be provided to patients, ultimately improving post-hospital compliance and overall patient satisfaction.

The development of standardized nursing safety management for day surgery remains inadequate. In this survey, only one-third of the day surgery nursing safety management indicators achieved an implementation rate of over 90%. To improve the safety management of day surgery nursing, it is imperative that medical institutions establish a day surgery center admission management system. This system is crucial for ensuring that patients’ physiological indicators meet the eligibility criteria for day surgery. The implementation of an admission criteria serves as a pivotal measure to reduce surgical risk and ensure patient safety (25, 26). The day surgery admission system should encompass the following: admission criteria for specific disease types and surgical procedures; for surgeons, surgical nurses, and anesthesiologists; and for patients. In particular, when formulating patient admission criteria, a comprehensive evaluation of the patient’s general condition, underlying diseases, and medical risks is required to determine suitability for day surgery. Secondly, it is recommended that medical institutions establish a specialized nursing pathway for day surgery. From 2016 to 2022, the proportion of day surgery in the total number of surgeries increased from 0.59 to 5.19%, and the number of specialized disciplines offering day surgery increased from 8 to 14 (27). The establishment of a specialized disease-specific nursing pathway for day surgery, along with the development of a department list, disease list, nursing list, surgical procedure list, and inspection list, was a way to facilitate integrated and standardized management of day surgery across various specialties and levels. Thirdly, it is recommended that medical institutions establish day surgery outpatient centers. Patients’ pre-admission preparation process involves numerous steps. Establishing a day surgery outpatient center facilitates improvements in the efficiency of and patient experience with outpatient care (28). Fourthly, the day ward and day surgery center are clearly demarcated, with safety signage that is highly visible and unambiguous. Our survey revealed that only 57.58% of institutions met the requirement. Clear physical separation and unambiguous signage are considered essential structural measures to prevent errors and enhance safety awareness among staff, patients, and visitors.

A survey assessing the demand for comprehensive management services among day surgery patients identified the requirement for a management platform as a key service expectation. This finding suggests a positive correlation between the quality of platform services and patient satisfaction (29). This study showed that 51.52% of medical institutions did not establish an electronic information system for day surgery, and 13.64% of medical institutions utilized intelligent platforms for post-discharge follow-up of day surgery patients. The implementation of standardized and information-driven management processes is essential for ensuring the quality of medical and nursing services, thereby facilitating the efficient and high-quality advancement of the day surgery model (30). With the advancement of smart management, information-based management, and mobile healthcare technologies, current research has developed a comprehensive management platform for day surgery patients, emphasizing health education, surgical consultation, and follow-up as its primary functions. This platform facilitates patients’ understanding of surgical procedures and associated knowledge, thereby effectively addressing their needs for information related to surgery, disease, and rehabilitation (31). Several scholars have employed various information technology tools, including mobile devices, interactive software, and artificial intelligence, to deliver preoperative health guidance and monitor postoperative rehabilitation adherence (32, 33). Jun et al. (34) have focused on health education as a pivotal element, devising a health education pathway based on key educational nodes throughout the diagnostic and treatment process for day surgery patients. They have established a health education knowledge base and developed an intelligent health education platform and patient health profiles utilizing artificial intelligence technology. This approach facilitated the delivery of precise and adaptable health education services, thereby enhancing patient satisfaction and optimizing the nursing service model. Future research should concentrate on the comprehensive integration of technologies like virtual reality and artificial intelligence to foster innovative applications and further develop the “Internet + Nursing Services” model in the field of day surgery management. Specifically, develop preoperative virtual reality simulation modules to assist patients in familiarizing themselves with the surgical environment and procedure. Additionally, deploy artificial intelligence-driven intelligent reminder systems for pre-hospital preparation to overcome the temporal and spatial barriers patients encounter during pre-hospital preparation and post-hospital rehabilitation. To alleviate the limitations of traditional day surgery health education models in terms of continuity, personalization, and interactivity, it is advisable to develop a comprehensive nursing service management platform. This platform should integrate three core functional modules: online one-on-one consultation for perioperative queries, real-time dynamic monitoring of postoperative vital signs and symptoms with intelligent early warning for abnormal indicators, and professional psychological support interventions. Meanwhile, establish a graphic interactive follow-up management system within the platform to conduct phased follow-up after discharge and push personalized rehabilitation guidance based on patients’ conditions, thereby enhancing patient satisfaction with medical service experiences and providing substantial support for the high-quality development of medical institutions.

## 5 Conclusion

Through a cross-sectional survey, this study provides the first systematic documentation of the current status of resource allocation, structural characteristics, service content and safety management practices in day surgery nursing services within Hubei Province, thereby laying an empirical foundation for optimizing regional day-surgery service models. Although day surgery has been progressively developing in China, management issues persist within medical institutions. Specifically, the proportion of day surgery among elective procedures and the utilization rate of medical resources in secondary healthcare institutions need improvement. Furthermore, the content of nursing services for day surgery requires enhancement, along with the promotion of continuous nursing models. The development of standardized nursing safety management systems for day surgery requires further development, and intelligent management approaches need to be further promoted.

A limitation is the use of convenience sampling, may introduce selection bias by over-representing hospitals with stronger management foundations. In addition, these findings offer preliminary insights into staffing patterns but should be interpreted with caution due to the partial data availability, and they may not be generalizable to all day surgery providers in the surveyed region. Future research should expand to include other regions, aiming to perform a comparative analysis of differences in the scale and implementation standards of day surgery across various medical institutions. Multi-center studies employing stratified or random sampling strategies would be valuable to validate and extend these findings.

## Data Availability

The original contributions presented in the study are included in the article/supplementary material, further inquiries can be directed to the corresponding author.
